# Altruism costs—the cheap signal from amygdala

**DOI:** 10.1093/scan/nst118

**Published:** 2013-08-24

**Authors:** Katarina Gospic, Marcus Sundberg, Johanna Maeder, Peter Fransson, Predrag Petrovic, Gunnar Isacsson, Anders Karlström, Martin Ingvar

**Affiliations:** ^1^MR Research Center and Osher Center for Integrative Medicine, Department of Clinical Neuroscience, Karolinska Institute, Retzius väg 8, 17177 Stockholm, ^2^KTH Royal Institute of Technology, Department of Transport Science, Teknikringen 10, 100 44 Stockholm and ^3^VTI, Transport Economics Unit, PO Box 920, 781 29 Borlänge, Sweden

**Keywords:** hypothetical bias, decision making, fMRI, amygdala

## Abstract

When people state their willingness to pay for something, the amount usually differs from the behavior in a real purchase situation. The discrepancy between a hypothetical answer and the real act is called hypothetical bias. We investigated neural processes of hypothetical bias regarding monetary donations to public goods using fMRI with the hypothesis that amygdala codes for real costs. Real decisions activated amygdala more than hypothetical decisions. This was observed for both accepted and rejected proposals. The more the subjects accepted real donation proposals the greater was the activity in rostral anterior cingulate cortex—a region known to control amygdala but also neural processing of the cost-benefit difference. The presentation of a charitable donation goal evoked an insula activity that predicted the later decision to donate. In conclusion, we have identified the neural mechanisms underlying real donation behavior, compatible with theories on hypothetical bias. Our findings imply that the emotional system has an important role in real decision making as it signals what kind of immediate cost and reward an outcome is associated with.

## INTRODUCTION

When people state their willingness to pay for something, the amount differs from the behavior when faced with a real purchase (Johannesson *et al.*, 1998). The systematic discrepancy between hypothetical and real choices is usually referred to as ‘hypothetical bias’ (Johansson-Stenman and Svedsäter, 2007; Kang *et al.*, 2011). In general, hypothetically derived estimates of the willingness to pay for something are higher than the corresponding willingness to pay derived from real choices (Murphy *et al.*, 2005). The underlying reasons to this phenomenon are complex. For example, real choices have costs for the decision maker; thus, when we make a real choice we have to deal with the direct comparison between a loss (e.g. money or time) and the gain (e.g. goods or favors). In contrast, hypothetical choices do not cost anything in real terms and we do not have to face any particular consequences (Kang *et al.*, 2011).

Private goods are products that have a market price, for example a cell phone or a pair of sneakers. As private goods have a known price for people, this gives a certain direction (anchor) for what the product is worth. In contrast public goods, like national parks and health programs do not have an obvious market price and our willingness to pay for these is more ambiguous (Carlsson and Martinsson, 2001). The degree of hypothetical bias differs for different kinds of goods, i.e. hypothetical bias tend to be greater for public goods than for private goods (Murphy *et al.*, 2005).

There is an abundant amount of research that shows that emotional processes affect decision making and economic decision making in particular (Bechara *et al.*, 2003; De Martino *et al.*, 2006; Knutson *et al.*, 2007; Gospic *et al.*, 2011). Both cortical and subcortical emotional structures affect choice behavior (De Martino *et al.*, 2006; Knutson *et al.*, 2007; Gospic *et al.*, 2011). For example, observing products that we like activates subcortical brain regions like nucleus accumbens (Knutson *et al.*, 2008) and cortical regions such as ventromedial prefrontal cortex (Paulus and Frank, 2003), both related to emotional processing in terms of ‘wanting’ and ‘liking’ (Berridge and Kringelbach, 2008; Kringelbach and Berridge, 2009). Activity in these regions also seems to be predictive of purchase (Knutson *et al.*, 2007). These findings are well aligned with the two-level model of decision making presented by Gläsher *et al.* (2010). The two-level model entail that model-free decisions have a limited amount of information and are made on a subcortical level. In contrast, model-based decisions have a richer presentation including future representation and are made on a cortical level (Gläscher *et al.*, 2010; Gospic *et al.*, 2011; Pezzulo and Rigoli, 2011).

It is plausible that real decisions should involve more emotional processing than hypothetical decisions as they are associated with real costs (FeldmanHall *et al.*, 2012). Amygdala is a subcortical structure that has been shown to be involved in emotional processing (LeDoux, 2007) and economic decision making (De Martino *et al.*, 2006; Gospic *et al.*, 2011). In a previous neuroeconomic study (Gospic *et al.*, 2011), we showed that instant social punishment in a real decision context was associated with amygdala activity. Thus, the rejection in the Ultimatum Game (Güth *et al.*, 1982) was viewed as a direct aggressive response mediated by a limbic structure (Gospic *et al.*, 2011). However, amygdala has also been shown to be involved in other decision processes such as in the framing effect (De Martino *et al.*, 2006; Roiser *et al.*, 2009). Both tasks involve instant aversive reactions that influence a decision. Moreover, amygdala participates in specific aspects of decisions such as risk (Brand *et al.*, 2007; Talmi *et al.*, 2009; Gupta *et al.*, 2011), regret (Coricelli *et al.*, 2007; Nicollea *et al.*, 2011), broken promises (Baumgartner *et al.*, 2009), monetary loss aversion (De Martino *et al.*, 2010) and cost input (Basten *et al.*, 2010; Li *et al.*, 2011). We therefore hypothesized that amygdala has an important role in all fast model-free decisions where an aversive element is present. Several studies on economic decision making have shown that decisions that are made over a longer time period (i.e. 4 s) involves cortical structures, e.g. insula, dorsolateral prefrontal cortex (dlPFC) and anterior cingulate cortex (ACC) (Sanfey *et al.*, 2003; Knoch *et al.*, 2006). These results could be generalized to that contemplated (hypothetical) decision could be considered as a model-based decision with a richer representation (Gläscher *et al.*, 2010; Gospic *et al.*, 2011; FeldmanHall *et al.*, 2012).

In the only brain imaging study, to our knowledge, on economical hypothetical bias, Kang *et al.* (2011) showed with a private goods, intra-individual design in males, that a cortical network consisting of medial orbitofrontal cortex (mOFC), ventral striatum and ACC was involved in both real and hypothetical decisions. The only discrepancy between real and hypothetical choices was that the network was more active in real decisions than in hypothetical decisions. The previous study by Kang *et al.* did not identify any neural structures that were specific for either real or hypothetical choices; neither did they detect any differences in subcortical emotional structures. This may be due to that Kang *et al.* had an experimental design that did not allow detection of a more rapid subcortical emotional response (Vuilleumier *et al.*, 2001; Gospic *et al.*, 2011) as their onset-time for when the choice was made varied from 0 to 4 s. Moreover, it may be questioned whether the intra-individual design can capture differences between real and hypothetical processes as the subjects are always influenced how they reacted in the other condition (Johansson-Stenman and Svedsäter, 2007). This may have precluded categorical differences between the two conditions. The current study aimed to separate neural structures involved in real *vs* hypothetical decisions and detect rapid, transient emotional processes in subcortical structures like the amygdala using a precise onset-time and a between group comparison.

**Fig. 1:**
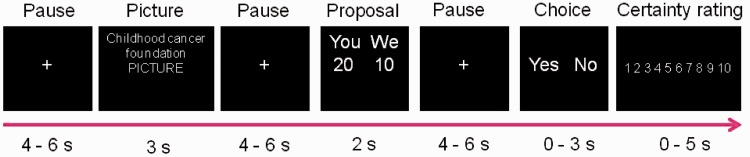
Experimental set-up. Participants were randomized to either the real or the hypothetical donation group. The task was either to reject or accept the proposals for a donation toward a charitable cause. The real group was informed that their decision could have a cost (if they chose to accept a proposal) while the hypothetical group was informed that they should answer according to how they would do if the paradigm was about real money. They were explicitly told that neither of their choices would cost them any real money. Control proposals were presented with a picture from one of the donation categories together with a text stating that: ‘this is not a proposal’. The onset-time of events used in the fMRI analysis were set to the onset of presentation of the picture, proposal and choice.

In the present study, subjects participated in a donation paradigm. Participants were randomly allocated to either a real or a hypothetical group and appropriately pre-informed. Participants were presented to monetary proposals that contained suggestions on how a proposed amount of money could be donated by themselves and together with different amounts of co-donations by us (the lab) to a charitable organization (Figure 1). The experimental task was either to accept or decline the presented proposal. The participants in the hypothetical group were instructed to answer as if they faced the proposal for real. The real group made decisions that were all potentially real, as one of the proposals were randomly selected and realized after the experiment, including both the donation from the subject as well as our co-donation (Carlsson and Martinsson, 2001). Moreover, our experimental design had a well-defined onset-time for when the choice was made, in order to detect rapid subcortical responses related to the choice situation (Gospic *et al.*, 2011). Based on the reasoning above, we hypothesized that real rapid decisions are subcortically driven (Gospic *et al.*, 2011), whereas contemplated hypothetical decisions are more cortically driven (Fuster, 2008) as they require a mental model representation of the imaginative outcome. Specifically, we predicted that amygdala is involved in the coding of cost and therefore, more present in real decisions. To override this cost signal i.e. to accept a costly proposal, this signal needs to be controlled by a regulatory network including rostral ACC (rACC) (Etkin *et al.*, 2006).

## BEHAVIORAL RESULTS

In order to establish the existence of a hypothetical bias in our experimental setting we investigated, with a mixed panel logit regression, if there were any discrepancies between real and hypothetical decisions. This model was used as donation behavior cannot *solely* be explained by group membership. The mixed logit model describes behavior in terms of the probability that an individual will accept a proposal and donate money. Furthermore, the probability is dependent on: stake level, donation, gender, hypothetical or real treatment, donation target and some interactions between these variables. The model is further explained in the statistical analysis section and regression results are displayed in Table 1.

### Hypothetical bias

First, across the pooled sample we could establish, with a likelihood ratio test, comparing models with and without treatment variables, that there was a hypothetical bias. That is we reject the hypothesis of no treatment effect: β*^r^* = β*^dr^* = β*^drm^* = *α_n_^rm^* = 0 (*Χ*^2^ = 23.2, df = 4, *P* = 0.0002).

**Fig. 2:**
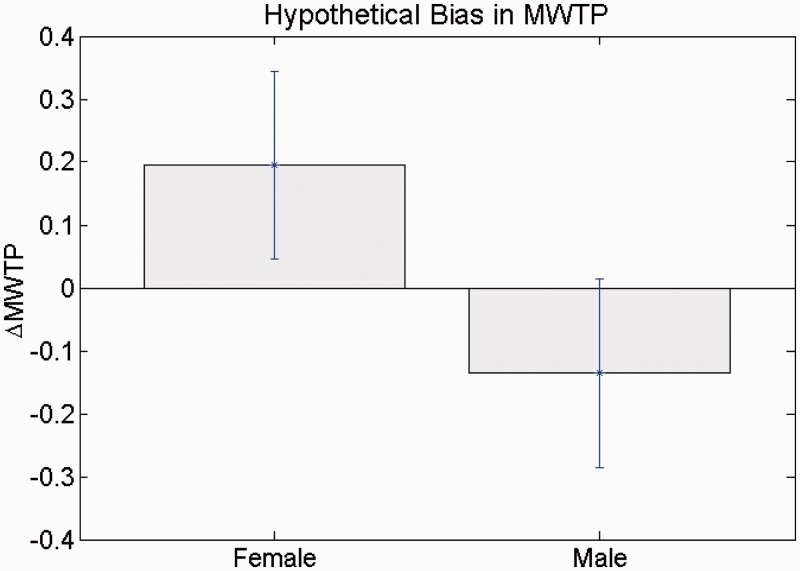
Hypothetical bias ± 2 s.e. The hypothetical bias is positive and significant for females (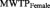 = 0.19, *P* = 0.009), negative and threshold significant for males (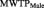 = −0.14, *P* = 0.070), and the difference in bias between the genders is significant (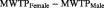 = 0.33, *P* = 0.003).

Second, to investigate how much each group was willing to donate, we compared marginal willingness to pay (MWTP) by dividing the parameters for ‘donation’ with the parameter for ‘you pay’. As the gender variable was significant in the mixed logit regression, we separated males and females when examining their MWTP. Females in the hypothetical group were willing to pay more money (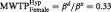) than females in the real group (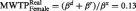). We reject the hypothesis of equal MWTP for females between the two groups as 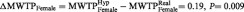 (Figure 2). Thus, females behaved according to the hypothetical bias phenomenon; females in the hypothetical group state a valuation 2.5 times higher than females in the real group. The same comparison for males showed a threshold significant result; males in the hypothetical group were willing to donate less money (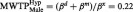) than males in the real group (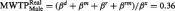). Testing the hypothesis of equal MWTP for males reveals a threshold significant difference (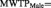 
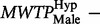
 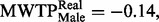 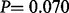) (Figure 2). Thus, males seemed to behave opposite to females as their hypothetical bias went in the other direction; the MWTP among males in the hypothetical group is only 0.6 times that of the males in the real group. In our sample, the difference in hypothetical bias between females and males is significant (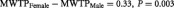
3) (Figure 2). We will return to this gender difference in the discussion.

### Stake level affects donation patterns

We hypothesized that stake level would affect donation patterns as higher stakes are associated with a greater cost for the participant. Stake level significantly affected donation behavior as the ‘you pay’ variable in the regression was significant (β*^x^* = −14.386, *P* = 0.000) (Table 1). That is, participants were less willing to donate e.g. 600 SEK than 50 SEK. Also, we made separate regressions for the hypothetical and the real group in order to establish if stake level affected both groups in a similar fashion. Indeed, stake level had a significant negative impact on donation behavior (*P* < 0.001) in both groups; that is, both groups accepted low stake proposals more frequently than high stake proposals.

**Table 1:** Mixed panel logit regression*.* You pay = the amount of money donated by the participant; donation = total of money donated; real = the real treatment group; male = male sex; interactions are indicated through multiplication, e.g. donation*real. Note that the reference donation target was ‘The Swedish Childhood Cancer Foundation’. The random coefficients *α_n_* and *α_n_^rm^* were assumed normally distributed and we estimated the mean and standard deviation of respective distribution. The mean of *α_n_^rm^* was not significantly different from zero

Coefficients	Est	SE	*t*	*Χ*^2^	*P*
β*^x^*—you pay	−14.386	2.664	−5.400	29.157	0.000
β*^d^*—donation	4.732	1.869	2.532	6.411	0.011
β*^dr^*—donation*real	−2.800	0.938	−2.986	8.916	0.003
β*^dm^*—donation*male	−1.524	0.966	−1.578	2.491	0.115
β*^drm^*—donation*real* male	4.752	1.339	3.549	12.593	0.000
β*^r^*—real	0.333	0.440	0.755	0.570	0.450
β*^m^*—male	−1.115	0.434	−2.570	6.604	0.010
β^2^—Stockholm City Mission	−1.628	0.268	−6.078	36.941	0.000
β^3^—Water Aid Sweden	−0.542	0.263	−2.065	4.263	0.039
β^4^—Save the Swedish Forest	−1.741	0.269	−6.474	41.908	0.000
β^5^—Doctors Without Borders	0.155	0.270	0.572	0.328	0.567
β^6^—Save the Rainforest	−0.883	0.265	−3.339	11.147	0.001
β^7^—Save the Seals	−1.596	0.269	−5.935	35.220	0.000
β^8^—Save the Tigers	−1.260	0.266	−4.747	22.535	0.000
Random coefficients					
Mean	Est	SE	*t*	*Χ*^2^	*P*
α_n_—intercept	3.017	0.389	7.762	60.253	0.000
s.d.	Est	SE	*t*	*Χ*^2^	*P*
α_n_—intercept	0.944	0.154	6.112	37.360	0.000
α_n_*^rm^*—real*male	1.889	0.574	3.292	10.834	0.001

Final LL = −801.0	LL0 = −1257.4			Nobs = 1814	

### Total amount of money donated affect the willingness to donate

In this study, we reinforced the participants’ willingness to donate by co-donations. That is, if the participant would donate 50 SEK we (the lab) would donate 10 SEK. One reason for this design was to give an imperative for the participant to donate money during the experiment and not after the experiment. This manipulation worked well as we achieved acceptance ratios of 0.59 and 0.53 for females under hypothetical and real treatments respectively, corresponding ratios for males were 0.37 and 0.48. The willingness to donate was positively affected by total donation levels for all, and significantly for males in the real group (β*^d^* + β*^m^* + β*^dr^* + β*^drm^* = 5.160, *P* = 0.007) and females in the hypothetical group (β*^d^* = 4.732, *P* = 0.011). Thus, these participants were more willing to donate 50 SEK themselves when the total donation was 80 SEK compared with 60 SEK.

### Charitable organization affects the willingness to donate

The willingness to donate was affected by charitable organization (Table 1). In the present sample, the most popular organizations to donate money to were: ‘Doctor Without Borders’, ‘The Swedish Childhood Cancer Foundation’ (reference organization in the model) and ‘Water Aid Sweden’ (Table 1).

## FMRI RESULTS

### Real decisions involve amygdala activation

**Fig. 3:**
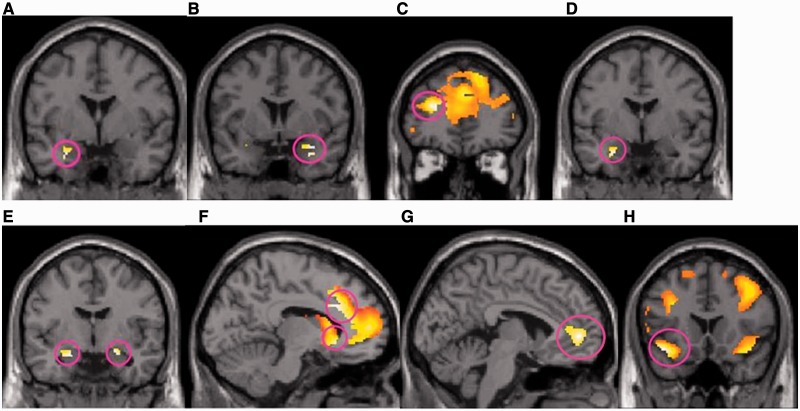
fMRI data showing activations related to the proposals, stake level, rejection and acceptance*.* (A) Proposals *vs* non-proposals in the real group, compared with the hypothetical group, yielded a higher activation in amygdala ([−28 0 −24] *Z* = 2.98, *P* = 0.042 voxel-level corrected). (B) Low stakes *vs* high stakes in the real group, compared with the hypothetical group, resulted in greater amygdala activation ([34 4 −20] *Z* = 2.79, *P* = 0.070 voxel-level corrected) and (C) dlPFC activation ([−26 42 20] *Z* = 3.45, *P* = 0.010 cluster-level corrected). (D) In the real group, compared with the hypothetical group, both rejected proposals, and (E) accepted proposals generated higher amygdala activity (rejected: [−26 −2 −24] *Z* = 3.32, *P* = 0.016 voxel-level corrected, accepted: left amygdala: [−20 −6 −18] *Z* = 2.91, *P* = 0.056 voxel-level corrected; right amygdala: [22 −4 −16] *Z* = 2.88, *P* = 0.060 voxel-level corrected). (F) In addition, accepted proposals in the real group resulted in greater activations in the ventral caudate ([−10 22 −6] *Z* = 4.03, *P* = 0.084 voxel-level corrected), and left ACC ([−10 22 30] *Z* = 4.59, 0.014 cluster-level corrected). (G) Subjects in the real group who accepted most donation proposals activated rACC ([ − 4 50 2] *Z* = 3.66, *P* = 0.037 cluster-level corrected) the strongest. (H) Insula activity ([−42 18 −14] *Z* = 3.94, *P* = 0.011 cluster-level corrected) was higher in the real group, compared with the hypothetical group, when participants viewed pictures that they later accepted.

We hypothesized that real decisions would involve more amygdala processing compared with hypothetical decisions; thus, our contrast of interest was: (proposals − non-proposals)_real group_ – (proposals − non-proposals)_hypothetical group_. Indeed, we detected a higher amygdala activity ([−28 0 −24] *Z* = 2.98, *P* = 0.042 voxel-level corrected) in the real group (Figure 3A). (See Supplementary Material for main effect of proposals.)

### Hypothetical decisions did not activate cortical areas more than real decisions

Our secondary hypothesis was that hypothetical decisions, compared with real decisions, would involve cortical processing to a greater extent. However, the contrast (proposals − non-proposals)_hypothetical group_ > (proposals − non-proposals)_real group_ did not result in any significant activation. Performing the same contrast in only females respectively males did not yield any significant results.

### Gender does not affect neural processing

As behavioral data implied that there was a gender effect in the hypothetical bias, we compared females with males in a factorial analysis. This analysis did not reveal any significant differences between genders. Exploratory gender analyses are presented in Supplementary Material and Figure S1.

### Stake level influence choice

As high stakes involve either a greater reward or a greater loss, in comparison to lower stakes, an interesting contrast would be to compare: (proposals_high stakes_ > proposals_low stakes_) _real group_ − (proposals_high stakes_ > proposals_low stakes_)_hypothetical group_. This contrast did not yield any significant results. In addition, the reverse contrast (proposals_low stakes_ > proposals_high stakes_) _real group_ − (proposals_low stakes_ > proposals_high stakes_)_hypothetical group_ was also of interest as participants in both groups accepted low stake proposals more frequently than high stake proposals. This comparison yielded a threshold significant activation in amygdala ([34 4 − 20] *Z* = 2.79, *P* = 0.070 voxel-level corrected) (Figure 3B). *Post hoc*, the same contrast with a cortical search volume showed a significant activation in the left dlPFC ([−26 42 20] *Z* = 3.45, *P* = 0.010 cluster-level corrected) (Figure 3C).

### Neural activation predicts the choice

Comparing (proposal_rejected_ > non-proposals)_real group_ − (proposal_rejected_ > non-proposals)_hypothetical group_ yielded significant amygdala activation ([−26 −2 −24] *Z* = 3.32, *P* = 0.016 voxel-level corrected) (Figure 3D). The contrast: (proposal_accepted_ > non-proposals)_real group_ − (proposal_accepted_ > non-proposals)_hypothetical group_ also yielded marginal significant activations in amygdala (left: [−20 −6 −18] *Z* = 2.91, *P* = 0.056 voxel-level corrected; right: [22 −4 −16] *Z* = 2.88, *P* = 0.060 voxel-level corrected) (Figure 3E). *Post hoc*, a greater activity was seen for accepted proposals in ventral caudate ([−10 22 −6] *Z* = 4.03, *P* = 0.084 voxel-level corrected) (Figure 3F) and left ACC ([−10 22 30] *Z* = 4.59, *P* = 0.014 cluster-level corrected) (Figure 3F). Thus, it seems that real choices, independently of acceptance or rejection, are associated with an increased amygdala processing. Complementary analyses are presented in Supplementary Material.

### Neural activity related to individual acceptance rate

We hypothesized that a prerequisite for acceptance is an increased cognitive control over the cost signal from amygdala. In order to examine the relationship between the top-down control network and acceptance rate, we performed a full factorial analysis where we included gender and treatment as factors and acceptance rate as a covariate in the proposals *vs* non-proposal condition. The subjects who accepted most donation proposals also activated rACC ([−4 50 2] *Z* = 3.66, *P* = 0.037 cluster-level corrected) (Figure 3G), specifically in the real group and independent of gender. We did not find any change in amygdala activity in the opposite contrast or any gender interactions.

### Neural response during picture viewing predicts acceptance

An important mechanism to understand is if a particular choice (i.e. ‘yes’ or ‘no’) is related to a certain neural processes before the actual choice has been made. To investigate the neural response that precedes ‘yes’ and ‘no’ we compared pictures_accepted_ > pictures_non-proposals_ and pictures_rejected_ > pictures_non-proposals_ between groups. The former analysis (pictures_accepted_ > pictures_non-proposals_)_real group_ − (pictures_accepted_ > pictures_non-proposals_) _hypothetical group_ revealed that subjects in the real group had a greater insula response ([−42 18 −14] *Z* = 3.94, *P* = 0.011 cluster-level corrected) (Figure 3H), than the hypothetical group, to pictures stating a proposal that they would later accept. The latter analysis did not reveal any significant neural responses related to rejection.

## DISCUSSION

The novel finding in the present study is that we show that there is a functional limbic involvement in real decisions, compatible with theories on hypothetical bias. Real donations activated the amygdala more than hypothetical donations (Figure 3A). Donations were common at low stake levels and at the low levels there was more expressed amygdala activation (Figure 3B). The actual act of donation (i.e. accepting proposals) was associated with amygdala activation and an increased activity in the caudate nucleus and rACC (Figure 3E and F). Interestingly, rejection of a donation proposal did only activate amygdala (Figure 3D). The comparison between hypothetical decisions *vs* real decisions did not reveal any differences. It should be noted that the timing in our paradigm was optimized to catch early responses in the limbic system.

The behavioral results revealed, as expected, that females were subject to a positive hypothetical bias (Figure 2), overstating their MWTP in the hypothetical context. Interestingly, the hypothetical bias for males was significantly lower than that of females, with a threshold result indicating even a negative bias (Figure 2). Other studies have found a gender difference in the opposite direction (e.g. Brown and Taylor, 2000), with a stronger positive bias in males than females. Yet, it has also been demonstrated that nationality may affect the sign of the hypothetical bias (Ehmke *et al.*, 2008). Another study, similar to ours, regarding donation behavior and hypothetical bias within a Swedish sample, revealed that it was the females that were prone to a strong positive bias (Carlsson *et al.*, 2010), while the results for males were inconclusive.

This study has yielded two arguments supporting that amygdala is more involved in emotionally salient decisions. First, facing real donation proposals, where real costs are projected, yielded a higher amygdala activity compared with hypothetical proposals (Figure 3A). Second, the frequency of donations was higher at low stake levels and this was associated with an increased activity in the amygdala (Figure 3B). Thus, this indicates that amygdala activity follows the actual monetary cost of donating rather than stake level per se.

The observation that amygdala was activated both for accepted and rejected proposals indicates that amygdala signals emotional salience (Figure 3D and E) (FeldmanHall *et al.*, 2012). It has been known for long, that amygdala represents both value of reward and aversive events in animals and humans (Hampton *et al.*, 2007; Smith *et al.*, 2009; Gupta *et al.*, 2011; Jenison *et al.*, 2011). The last decade, research has shown that amygdala also represents aversive components contributing to decision processing such as risk (Brand *et al.*, 2007; Talmi *et al.*, 2009; Gupta *et al.*, 2011), regret (Coricelli *et al.*, 2007; Nicollea *et al.*, 2011), broken promises (Baumgartner *et al.*, 2009) and monetary loss aversion (De Martino *et al.*, 2010). This would be in line with the emotional cost that is associated with real decisions *vs* hypothetical decisions in the present experiment. Notably, it is not possible to distinguish whether the signal reflects pure salience (cost) or arousal.

It has been suggested that the cost component mediated by the amygdala is added to a benefit component mediated by the ventral striatum in order to make a decision (Basten *et al.*, 2010; Li *et al.*, 2011). The inputs of cost and benefit seem to be integrated in the ventromedial prefrontal cortex (Hampton *et al.*, 2007; Basten *et al.*, 2010; Li *et al.*, 2011). Interestingly, caudate activation was present in the real group during acceptance but not rejection (Figure 3F). In line with the presented studies above, this pattern of co-activation may be interpreted as amygdala codes for the ‘cost’ of donation, whereas the caudate may code for the reward component (de Quervain *et al.*, 2004; Harbaugh *et al.*, 2007; Basten *et al.*, 2010; Li *et al.*, 2011).

To make a decision it is required to weigh cost *vs* reward by computing a cost-benefit difference. It has been proposed that this computation is performed in vmPFC/rACC (Basten *et al.*, 2010). The observed ACC activity (Figure 3F) in the condition where subjects accepted proposals may reflect such a computation. However, this computation may not be sufficient when the cost is high, and a peer-pressure to choose the costly alternative is present. In this situation, the cost signal from amygdala must be overridden by top-down regulatory mechanisms. rACC has been shown to be important to control emotionally salient amygdala activity that interferes with a task (Etkin *et al.*, 2006). We suggest that the rACC activity in the present study reflects such a control. In line with this idea, we observed that the subjects who accepted more proposals also showed stronger rACC activation in the real group (Figure 3G). In our exploratory analyses, we investigated neural correlates in males that could explain their unexpected behavior. We noted that men accepted more proposals in the real group and concomitantly activated rACC. *Post hoc*, a psychophysiological interaction (PPI) analysis revealed a functional connectivity between amygdala and rACC in males (see Supplementary Material and Figure S2). This is suggestive that there is a difference between males and females. However, this result needs verification in an independent sample. Extrapolating this line of thoughts to the general results, the observed co-activation of caudate and ACC for accepted proposals may be interpreted as these structures help to override the ‘cheap’ signal from amygdala. This concept is in agreement with rACC involvement in pro-social behavior (FeldmanHall *et al.*, 2012).

In the real group, we observed that the act of donation was predicted by an increased insular activity during the initial picture presentation of the charitable organization (Figure 3H). This insular activity could reflect an expression of empathy. For example, in a series of studies Singer *et al.* (2006) has shown that insula activity is involved in neural processing of empathy. Moreover, it has also been demonstrated that insula activity predicts costly helping of others (Hein *et al.*, 2010; FeldmanHall *et al.*, 2012). Thus, our finding is well in line with previous literature that suggests that insula is important for empathic processing.

We observed dlPFC activations in the real group in response to stake level (Figure 3C); this finding is in agreement with previous studies (Sanfey *et al.*, 2003; Knoch *et al.*, 2006; Wright *et al.*, 2011) that imply that dlPFC processes contextual information. An alternative interpretation could be that dlPFC activation represents willingness to pay (Plassmann *et al.*, 2007). However, willingness to pay could also be seen as part of contextual information processing as the action of evaluating what goods are worth requires upholding of explicit values (Camus *et al.*, 2009).

The greatest difference between the present study and the study by Kang *et al.* is that the present study detected amygdala responses. We suggest that this difference is mainly due to the construction of our paradigm. Previous fMRI studies have shown that timing is crucial to detect transient amygdala responses (Vuilleumier *et al.*, 2001; Gospic *et al.*, 2011). Another important aspect was that their study had an intra-individual design, possibly precluding a real difference between hypothetical and real decisions since they may interact (Johansson-Stenman and Svedsäter, 2008). This design feature also contributes to internal consistency, i.e. the strive for consistency between attitude and behavior (Johansson-Stenman and Svedsäter, 2008). The present study *vs* Kang *et al.* is also different in which sex that was studied, if private or public goods were used and within/between group comparisons. All these factors have shown to be important for inducing hypothetical bias and donation behavior (Brown-Kruse and Hummels, 1993; Blackburn *et al.*, 1994; Murphy *et al.*, 2005; Carlsson *et al.*, 2008; Johansson-Stenman and Svedsäter, 2008).

In conclusion, we have segregated the neural mechanisms involved in real *vs* hypothetical choice behavior. Our main finding is that there is a functional limbic involvement in real donation behavior, compatible with hypothetical bias theories. This implies that the emotional system has an important role in real decision making as it signals what kind of cost and reward an outcome is associated with.

## EXPERIMENTAL PROCEDURES

### Subjects

A total of 38 right-handed, healthy, volunteers with the mean age of (24.34 ± 2.92 years) (10 males in the real group, 9 males in the hypothetical group, 9 females in the real group and 10 females in the hypothetical group) were included in the data analyses. All participants gave their informed consent in writing. The study was approved by the governmental regional ethical review board in Stockholm, Sweden. (For more details see Supplementary Material.)

### Stimuli

Each subject was shown 60 different emotional pictures (Figure 1). Each picture was novel and was either pleasant or unpleasant. All proposals had the exact same wording, except for the total stakes that varied. Subjects were instructed to respond with either ‘yes’ or ‘no’ to the proposals by pressing a button. In the neutral control condition, the subjects were shown a picture with the text ‘no proposal’ and subjects were instructed to respond ‘no’ to these. There were six different stake levels for the proposals and subjects were shown 48 proposals and 12 neutral messages (non-proposals).

In the real group, every proposal had a 1/60 chance to be realized in terms of real money. However, if the subjects chose to reject an offer no money would be donated to the charity if that particular proposal was chosen to be realized. In the end of the study, donations to the respective charity organizations were made anonymously. After the first two proposal sequences in each session a certainty rating was displayed and participants were asked to rate how secure they felt of their choice.

The onset-time when the proposals were shown were included as regressors of interest (individual regressors for when the subject accepted, rejected and the control condition) in the subsequent general linear model (GLM) analysis of the fMRI analysis.

### Charitable organizations and picture selection

We chose eight well-known charitable organizations in Sweden and balanced the content between arousal, valances, causes, facial content and complexity.

### Monetary reward

All subjects were instructed that they would get 1000 SEK (148 USD) for participating in the experiment. The participants in the real group were informed that they could have a cost if they choose to donate money in the experiment. In contrast, the hypothetical subjects were instructed that they only played with hypothetical money.

Following the fMRI experiment, hypothetical subjects were reinformed and got to play a real game according to a similar procedure as the real group had done. This procedure was performed in order to account for reimbursement fairness and to allow all participants to receive exactly the same information prior to the experiment. After the experiment, every subject made an oral promise to not reveal the experimental manipulation to their peers.

### Experimental procedures

Upon arrival, subjects were randomly assigned to either the hypothetical group or the real group. The subjects were asked to read the instructions for the experiment and their understanding of the experimental task was checked with a questionnaire. All subjects passed this test.

The order in which the pictures and proposals were presented was randomized in advance and all subjects underwent two scanning sessions and each session contained 30 pictures/proposals. We used an fMRI-compatible mouse in the scanner to register the subjects’ responses. After the scanning was completed, subjects were asked to answer some questions. Moreover, the subjects in the hypothetical group were asked to perform a ‘real’ donation game.

### Statistical analysis

#### Behavioral data

Behavioral data was analyzed using a mixed panel logit model. The model is formulated through the latent variable *V*_in_, where:

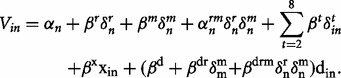

Behavior is modeled as the response *y*_in_, which is given by:

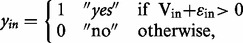

Where the unobservable *ε*_in_ are assumed independent and identically distributed (i.i.d.) logistic. Due to the unobserved factors in the responses, we model the probabilities of donation as *P*_in_ = Pr(*y*_in_ = 1). Using these probabilities, we estimate the parameters of our model through simulated maximum likelihood.

*V*_in_ may be interpreted as the utility of responding ‘yes’ to proposal *i*, for individual n, this interpretation is frequently used in random utility models (RUMs). The mixing parameters *α_n_* and *α_n_^rm^* denotes random taste variations (basic tendency to donate), which are assumed normally distributed over individuals. The shift parameters β*^r^*, β*^m^* and β*^t^* describe the shifts in utility for the real group, males and the different donation targets. The marginal disutility of spending money is given by β*^x^*, which reflects the behavioral sensitivity to the stake level *x*_in_ (you pay). The total donation in a proposal is denoted *d*_in_. Gender and treatment are represented by the indicator variables *δ^m^* and *δ^r^*. Thus, we have the marginal utility of total donations for hypothetical decisions - females β*^d^*, real decisions females β*^d^* + β*^dr^*, hypothetical decisions - males β*^d^* + β*^dm^* and real decisions males β*^d^* + β*^dr^* + β*^dm^* + β*^drm^*. The MWTP for these four categories is simply given by the quota between the marginal utility of donations and the marginal utility of spending money. Statistical tests regarding the MWTP are performed using the delta method. The model structure is similar to conventional logit models used for eliciting hypothetical bias in choice experiments (Johansson-Stenman and Svedsäter, 2008).

### fMRI data

#### Image acquisition

We used a GE-3.0 T MR-scanner to measure the blood-oxygen level-dependent (BOLD) responses. A T2* − weighted echoplanar image (EPI) sequence was applied. The following protocol was used: number of slices: 36, slice thickness: 4.5 mm, interslice gap: 0.5 mm, field of view (FOV): 220 × 220 mm, time echo (TE): 40 ms and time repetition (TR): 2.5 s. In all, 168 and 161 image volumes were acquired during the two scanning sessions, respectively. In addition, we acquired an anatomical T1-weighted 3D image volume from each subject (3D-SPGR, TR/TE = 35/6 ms, flip = 35 deg, 124 coronal images, matrix size (0.9 × 1.0 × 0.9 mm^3^).

#### Image analysis

##### Pre-processing

The functional MRI data were analyzed with the SPM8 software (http://www.fil.ion.ucl.ac.uk/spm/software/). The following pre-processing steps were performed: realignment, co-registration and normalization with respect to the MNI-compatible EPI template provided in SPM8. Finally, spatial smoothing was performed with a Gaussian kernel of 10 mm full-with-half-maximum (FWHM). Event onset-times pertaining to the proposals and control conditions were convolved the canonical hemodynamic response function as implemented in SPM8 and inserted into a GLM. We specified four different models were we included regressors of interest. In the main model, we included four regressors for each scanning session: (i) picture onset, (ii) proposal onset, (iii) non-proposal (control condition) and (iv) reaction time. (See Supplementary Material for more detailed specifications on the other three models.) We corrected for residual movement-related variance in the data by including six motion parameters in the model. High-pass filtering (cut-off frequency = 128 s) was used to remove low-frequency noise.

##### Regions of interest

All masks used in the second-level analyses were created with the wfu_pickatlas (Maldjian *et al.*, 2003, 2004) tool in SPM8. To validate our study design (timing), we made a global search in the contrast proposals > non-proposals. We included the following 13 regions of interests (ROI) (bilaterally): ACC, insula, caudate, putamen, mOFC, inferior orbitofrontal cortex, superior orbitofrontal cortex, rectus, superior medial frontal cortex, superior frontal cortex, medial frontal cortex, inferior frontal operculum and inferior frontal triangularis. In addition, we analyzed amygdala activity with a separate ROI. We used a mask to reduce the search volume and the included regions were selected based on present literature (Sanfey *et al.*, 2003; de Quervain *et al.*, 2004; Rilling *et al.*, 2004; Gospic *et al.*, 2011). This reduced the search volume to ¼.

To answer our primary hypotheses, we used a bilateral amygdala mask in the interaction contrasts when comparing the real group *vs* the hypothetical group. Our amygdala hypotheses were based on previous literature that shows that amygdala is important for decision making and participates in real rapid decisions (De Martino *et al.*, 2006; Gospic *et al.*, 2011). In the extended *post hoc* analyses, the thirteen above stated regions were used for global search in the contrasts were only the amygdala mask had been used initially. The opposite strategy was used for comparisons between the hypothetical group *vs* the real group; i.e. then we used the 13-area mask first and then the bilateral amygdala mask. We had hypothesized that cortical regions like the insula, dlPFC and ACC would be more important for hypothetical decisions (Sanfey *et al.*, 2003; Knoch *et al.*, 2006; Fuster, 2008). Striatal areas were included as a previous study has shown that they are important for decision making (de Quervain *et al.*, 2004).

We hypothesized that amygdala activity must be suppressed by top-down regulatory processes in order to accept costly proposals. Therefore, we defined a search volume including rACC, Obfc, vmPFC, dlPFC to study how acceptance rate variability in different subjects relates to proposals (included regions in the wfu_pickatlas: ACC, mOFC, mid orbitofrontal cortex, inferior orbitofrontal cortex, superior orbitofrontal cortex, rectus, superior medial frontal cortex, superior frontal cortex, medial frontal cortex, inferior frontal operculum and inferior frontal triangularis).

In the general PPI analyses, we used the 14-region mask but excluded the left amygdala, as this region was our seed region.

##### Reporting results

The SPM [T] map threshold was set to *P* < 0.05 (uncorrected) in all contrasts. We choose this threshold as amygdala’s search region is small. However, as our hypotheses are well defined and we only report corrected results this approach is statistically valid. To be consistent, we applied the same threshold for the cortical search volume. Our amygdala results were never below 43 voxels and results from cortical searchers were 976 voxels or more. This indicates that our results have not occurred by chance. All results are reported as voxel-level corrected, unless otherwise stated (i.e. cluster-level corrected). Details about specific statistics for our comparisons are specified in Supplementary Table S1.

## SUPPLEMENTARY DATA

Supplementary data are available at *SCAN* online.

Supplementary Data
