# Supplementary material for: Altruism costs—the cheap signal from amygdala
Source: Soc Cogn Affect Neurosci. 2013 Aug 24;9(9):1325–32. doi: 10.1093/scan/nst118 (PMC4158368; doi:10.1093/scan/nst118)
Supplement: Supplementary Data [file supp_9_9_1325__index.html]

Altruism Costs - the Cheap Signal from Amygdala — Altruism costs—the cheap signal from amygdala — Altruism costs—the cheap signal from amygdala — Supplementary Data 

# Altruism costs—the cheap signal from amygdala

## Supplementary Data

files

**Files in this Data Supplement:**

- Supplementary Data - doc file
- Supplementary Data - tif file
- Supplementary Data - tif file
- Supplementary Data - xls file
