# Supplementary material for: Altruism costs—the cheap signal from amygdala
Source: Soc Cogn Affect Neurosci. 2013 Aug 24;9(9):1325–32. doi: 10.1093/scan/nst118 (PMC4158368; doi:10.1093/scan/nst118)
Supplement: Supplementary Data [file supp_nst118_scan-12-221-File006.doc]

# Supplementary material

## FMRI Results

### Main effect of proposals

To validate our study design we examined the main effect of proposals (proposals > non-proposals) in within group comparisons. In the real group this contrast yielded activation in dACC (left superior medial gyrus) ([-6 20 40] Z = 4.52, p = 0.001 cluster-level corrected) (Table S1); the corresponding contrast in the hypothetical group showed no significant activations.

### Neural activation predicts the choice

To investigate if neural response patterns could predict acceptance or rejection of proposals we compared: proposals _accepted_ > proposals _rejected_ between groups (real versus hypothetical) and vice versa. These contrasts did not yield any significant results.

### Gender analyses

As behavioral data indicated gender differences, we performed some exploratory analyses to analyze this more thoroughly. When comparing (proposals – non-proposals) _real group females_ – (proposals – non-proposals) _hypothetical group females_ we observed an amygdala activation (left: [-24 -2 -26] Z = 3.25, p = 0.024 voxel-level corrected) (Fig. S1A), similar to the activation observed in the interaction contrast over the whole group. The corresponding contrast in males did not yield any significant amygdala result. However, the post hoc analysis, including a cortical search volume, showed that males in the real group expressed greater rACC activity (left: [-6 52 4] Z = 4.30, p = 0.040 voxel-level corrected) (Fig. S1B) compared to the males in the hypothetical group.

### Psychophysiological interaction

Surprisingly, males behaved opposite to the hypothesis by accepting more offers in the real group compared to the hypothetical group. To further investigate this finding we performed post hoc psychophysiological interaction (PPI) analyses. We hypothesized that males would have a greater cognitive control of the cost signal from amygdala or that they would be more emotionally engaged. We used left amygdala as a seed region and the coordinates from the proposal versus non-proposal analysis (MNI coordinates: [-28 0 -24]).

In the one sample t-test for males, in the real group, we detected significant activity in the insula ([-36 20 6] Z = 4.83, p = 0.016 voxel level corrected) (Fig. S2A) and the rACC ([18 54 0] Z = 4.63, p = 0.032 voxel level corrected) (Fig. S2B). The corresponding analysis for females did not yield any significant results. A comparison between real group males versus hypothetical group males revealed a significant result in insula ([-46 36 18] Z = 4.63, p = 0.000 cluster level corrected) (Fig. S2C). Lastly, we compared males in the real group to females in the real group; this comparison also yielded a significant result in insula ([-34 20 8] Z = 4.37, p = 0.029 voxel level corrected) (Fig. S2D).

This shows that there was a functional relation between amygdala, and insula and rACC, only in males (Fig. S2A to S2D).

## Methods

### Subjects

40 right handed, healthy, volunteers with the mean age of 24.18 ± 2.95 years (mean ± stdv) (20 males, 20 females) were included in the study. Due to technical problems the data for two subjects were lost; thus, 38 subjects were included in the data analyses. Subjects had no prior or present history of psychiatric illness or neurological disease. All subjects were healthy and took no medications with the exception of birth control pills and mild allergic medications.

### Stake levels

The following six stake levels were presented in the study: 20, 50, 100, 200, 300, and 600 yielding a total of 18 different kinds of combinations. The different combinations of stakes were (you donate/we donate (in SEK)): 20/5, 20/10, 20/20, 50/10, 50/20, 50/30, 100/20, 100/40, 100/60, 200/40, 200/80, 200/120, 300/60, 300/120, 300/180, 600/120 600/240 600/360.

### Stimulus timing

Before each picture the subject was shown a resting frame containing a hair cross, for a duration that was randomized between 4 to 6 seconds. Thereafter, a picture with a charity organization was displayed for 3 seconds. The picture was followed by a jittered pause (4 to 6 seconds) and the proposal. The proposal sign (presented for 2 seconds) stated how much money they could donate respectively how much money we (the lab) would donate. The proposal was followed by a jittered pause (4 to 6 seconds) and then a sign stating: “Yes” and “No” was shown. The “Yes” and “No” sign lasted until a choice had been made or for 3 seconds. During the “Yes” and “No” sign the subjects had been instructed either accept or reject the donation proposal by either pressing “Yes” with their index finger or “No” with their ring finger. These certainty rating frame lasted until a choice had been made or for 5 seconds. A total of four certainty ratings were collected during the whole experiment.

### Picture selection

Eight donation targets were presented in the study, four humanitarian and four environmental. The four humanitarian organizations were: Swedish Childhood Cancer Foundation, Stockholm City Mission, Doctors Without Borders and Water Aid Sweden. The four environmental organizations were: Urskog 2000 (“save the Swedish forest”), Vi-Skogen (“save the rainforest”), Save the Seals in the Baltic Sea (World Wide Fund for Nature (WWF)) and Save the Tigers (WWF). A total of 208 pictures were depicted from the internet by the experimenters. Half of these were unpleasant and half were pleasant. All pictures were independently rated according to the IAPS convention by three of the experimenters. The four pictures that were rated the highest respectively the lowest in valance were depicted from each category with the respect to also balance the material for arousal, faces and complexity. The average arousal for the categories were; 6.7 ± 0.44 for the pleasant pictures and 2.5 ± 8.4 for the unpleasant pictures.

### Experimental procedures

Each protocol was presented twice, one time for a subject in the hypothetical group, and one time for a subject in the real group.

Subjects responded “yes” by pressing a key with their index finger and “no” by pressing a key with their ring finger. Their middle finger was used to scroll when performing the certainty rating which was present after the two first pictures in each trial. Hence, the certainty measure was collected four times in total and interpreted as a subject’s “trait.”

### Fmri data

The main contrasts of interest when analyzing group data were the following: First, we compared proposals > non-proposals in a one sample t-test including all subjects in respectively treatment group to ensure consistency. As a follow-up we performed an interaction analysis comparing (proposals – non-proposals) _real group_ – (proposals – non- proposals) _hypothetical group_ and v.v. i.e. hypothetical group > real group. The interaction analysis was also performed in only females respectively males.

Second, to detect possible gender differences we performed a factor analysis where treatment and gender were included as factors. Third, to study the influence of stake levels we compared (proposals _high stakes_ > proposals _low stakes_) _real group_ - (proposals _high stakes_ > proposals _low stakes_) _hypothetical group_ and (proposals _low stakes_ > proposals _high stakes_) _real group_ - (proposals _low stakes_ > proposals _high stakes_) _hypothetical group_. Both these analysis were also performed comparing the hypothetical group versus the real group.

Fourth, to investigate the effect of acceptance and rejection we performed the following analyses both within and between groups (real > hypothetical and hypothetical > real): (proposals _accepted_ > proposals _rejected_), (proposals _rejected_ > proposals _accepted_), (proposal _accepted_ > non- proposals) and (proposal _rejected_ > non-proposals). In a related analysis we wanted to understand how individuals that have a higher acceptance rate differ (in proposals > non-proposals) from those with lower acceptance rate. Especially, we were interested if these subjects showed a stronger top-down regulation of the cost input from amygdala. Therefore, we used the acceptance rate as a regression variable in a full factorial analysis where the other factors were real group and gender.

Fifth, we compared (pictures _accepted_ > pictures _non-proposals_) and (pictures _rejected_ > pictures _non-proposals_) to examine if a particular choice (i.e. “yes” or “no”) was related to a certain neural processes before the actual choice had been made.

## Image analysis

### Pre-processing

In addition to the main model we also used the following three models. To investigate the effect of stake level we divided the proposal variable in to two separate variables: high stake proposals (200, 300, 600 SEK) and low stake proposals (20, 50, 100 SEK). In the analyses were we examined the effect of acceptance/rejection we divided the proposal variable in to two separate variables: accepted proposals (“Yes”) and rejected proposals (“No”). Moreover, the contrasts were we analyzed if acceptance/rejection could predict picture processing we divided the picture variable in to two separate variables: pictures that were followed by a “yes” and pictures that were followed by a “no.”

### Psychophysiological interaction

Post hoc, we investigate with psychophysiological interaction (PPI) analyses, if there were any functional anatomical correlates to the previous detected behavioral differences between sexes. We used left amygdala as a seed region and the coordinates from the proposal versus non-proposal analysis (MNI coordinates: [-28 0 -24]). Both within group analyses and between group analyses were performed.

## SUPPLEMENTARY FIGURE LEGENDS

**Figure S1. fMRI data related to gender.** (A) Proposals in the real group in females, compared to the hypothetical group in females, also yielded a higher amygdala activity ([-24 -2 -26] Z = 3.25, p = 0.024 voxel-level corrected). (B) However, proposals in the real group in males, compared to the hypothetical group in males, resulted in a higher rACC activation (left: [-6 52 4] Z = 4.30, p = 0.040 voxel-level corrected).

**Figure S2. Psychophysiological interactions.** (A) For males in the real group, left amygdala activity co-varied with insula activity ([-36 20 6] Z = 4.83, p = 0.016 voxel level corrected), and (B) the rACC ([18 54 0] Z = 4.63, p = 0.032 voxel level corrected). (C) A comparison between real group males versus hypothetical group males revealed a significant co-variation in insula ([-46 36 18] Z = 4.63, p = 0.000 cluster level corrected). (D) Similarly, the corresponding comparison between males in the real group versus females in the real group also yielded a significant result in insula ([-34 20 8] Z = 4.37, p = 0.029).

## SUPPLEMENTARY Table LEGEND

**Table S1. Cerebral foci of activation related to real and hypothetical decisions.** p = proposals; np = non-proposals; real = real group; hypothetical = hypothetical group; lp = low stake proposals; hp = high stake proposals; pr = proposals that were rejected; pa = proposals that were accepted; pia = pictures that were accepted; pnp = pictures that were non-proposals; dlPFC = dorsolateral prefrontal cortex; d/rACC = dorsal/rostral anterior cingulate cortex; Hemi = hemisphere; R = right; L = left.
