# Supplementary figures and images for: Altruism costs—the cheap signal from amygdala
Source: Soc Cogn Affect Neurosci. 2013 Aug 24;9(9):1325–32. doi: 10.1093/scan/nst118 (PMC4158368; doi:10.1093/scan/nst118)

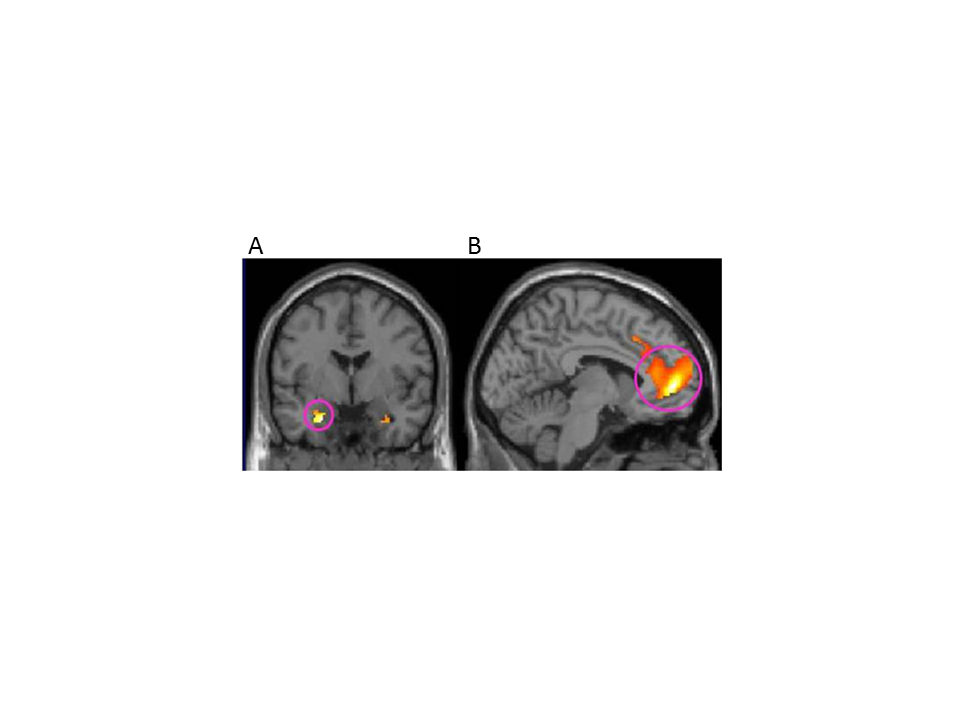

Supplement: Supplementary Data [file supp_nst118_scan-12-221-File007.tif]

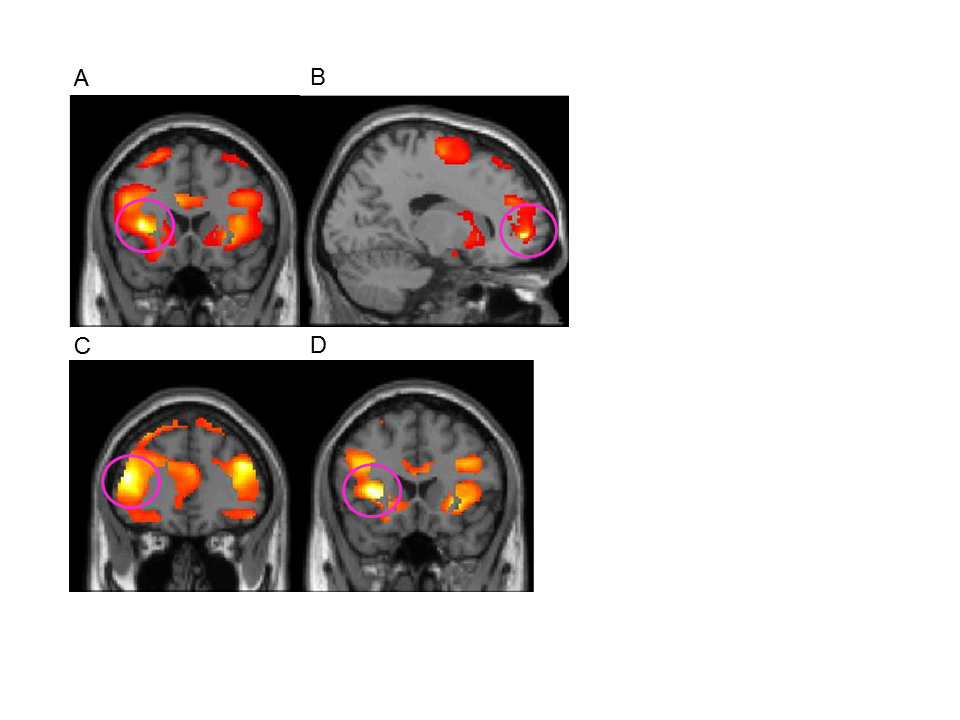

Supplement: Supplementary Data [file supp_nst118_scan-12-221-File008.tif]
